# Immunity to human endogenous retroviruses

**DOI:** 10.1186/1742-4690-8-S2-O19

**Published:** 2011-10-03

**Authors:** Douglas F Nixon

**Affiliations:** 1 University of California, San Francisco, USA

## 

Human endogenous retroelements (which rely on an RNA intermediate before integration into the genome) can be classified into the non-long terminal repeat (LTR) class and the LTR class, represented by human endogenous retroviruses (HERV). Many HERV entered the primate germ line as infectious retroviruses at several time points during human evolution. Out of the six HERVsuperfamilies, HERV-K (HML-2) is considered to be the youngest and most transcriptionally active. Various studies have identified adaptive immune responses to HERV under certain disease conditions, but whether these represent beneficial or deleterious immune responses is unclear. We have focused on the study of adaptive immunity to HERV in the context of HIV infection, and will present data on the cell mediated and humoral responses to certain HERV families. We find that HERV specific T cell responses can be found at high frequency in certain HIV infected subjects, and specific antibody responses are also identified. The functional activities of these adaptive immune mechanisms may play a role in HIV pathogenesis.

